# Phytochemicals With Anti 5-alpha-reductase Activity: A Prospective For Prostate Cancer Treatment

**DOI:** 10.12688/f1000research.51066.3

**Published:** 2021-07-06

**Authors:** Aziemah Azizi, Nuramalina H Mumin, Naeem Shafqat

**Affiliations:** 1 PAPRSB Institute of Health Sciences, Universiti Brunei Darussalam, Jalan Tungku Link, Gadong, BE1410, Brunei

**Keywords:** 5-alpha-reductase, Testosterone, Dihydrotestosterone, Finasteride, Dutasteride, Phytochemicals, Phytosterols, Polyphenols, Androgens, Prostate cancer

## Abstract

Prostate cancer (CaP) is one of the leading causes of death in men worldwide. Much attention has been given on its prevention and treatment strategies, including targeting the regulation of 5-alpha-Reductase (5αR) enzyme activity, aimed to limit the progression of CaP by inhibiting the conversion of potent androgen dihydrotestosterone from testosterone that is thought to play a role in pathogenesis of CaP, by using the 5-alpha-Reductase inhibitors (5αRis) such as finasteride and dutasteride. However, 5αRis are reported to exhibit numerous adverse side effects, for instance erectile dysfunction, ejaculatory dysfunction and loss of libido. This has led to a surge of interest on plant-derived alternatives that might offer favourable side effects and less toxic profiles. Phytochemicals from plants are shown to exhibit numerous medicinal properties in various studies targeting many major illnesses including CaP. Therefore, in this review, we aim to discuss the use of phytochemicals namely phytosterols, polyphenols and fatty acids, found in various plants with proven anti-CaP properties, as an alternative herbal CaP medicines as well as to outline their inhibitory activities on 5αRs isozymes based on their structural similarities with current 5αRis as part of CaP treatment approaches.

## Introduction

Prostate cancer (CaP) is the second most deadly malignancy in men after lung cancer and the fifth leading cause of death worldwide, accounting for 7.1% (1,276,106) of the new cases and 3.8% (358,989) of total death in males in 2018 ( Rawla, 2019). According to the United Kingdom Cancer Research Centre, over 47,500 men are diagnosed with CaP each year, where one man dies from it every 45 minutes. CaP is also estimated to be the most common cancer by 2030, as one in eight men destined to be diagnosed with CaP in their lifetime. CaP is a malignant tumour that is caused by unregulated prostate cell division resulting in an abnormal cellular growth that leads to a potential spread of cancer to other body parts ( Ochwang’i *et al.*, 2014; Packer & Maitland, 2016). The current primary treatments for CaP are surgery, radiation therapy, proton beam therapy, chemotherapy, cryosurgery, high intensity focused ultra-sound and hormonal therapy, depending on the clinical conditions, outcomes and disease progression among others ( Chen & Zhao, 2013). The latter strategy was largely anticipated, considering CaP is a hormones-driven disease especially during the initial stage ( Taplin *et al.*, 1995). Therefore, targeting the hormones involved in the CaP’s pathway mechanisms seems to be a potentially useful approach in developing CaP prevention and treatment strategies.

### Androgens and 5-alpha-Reductase enzymes (5αRs)

**Figure 1.**
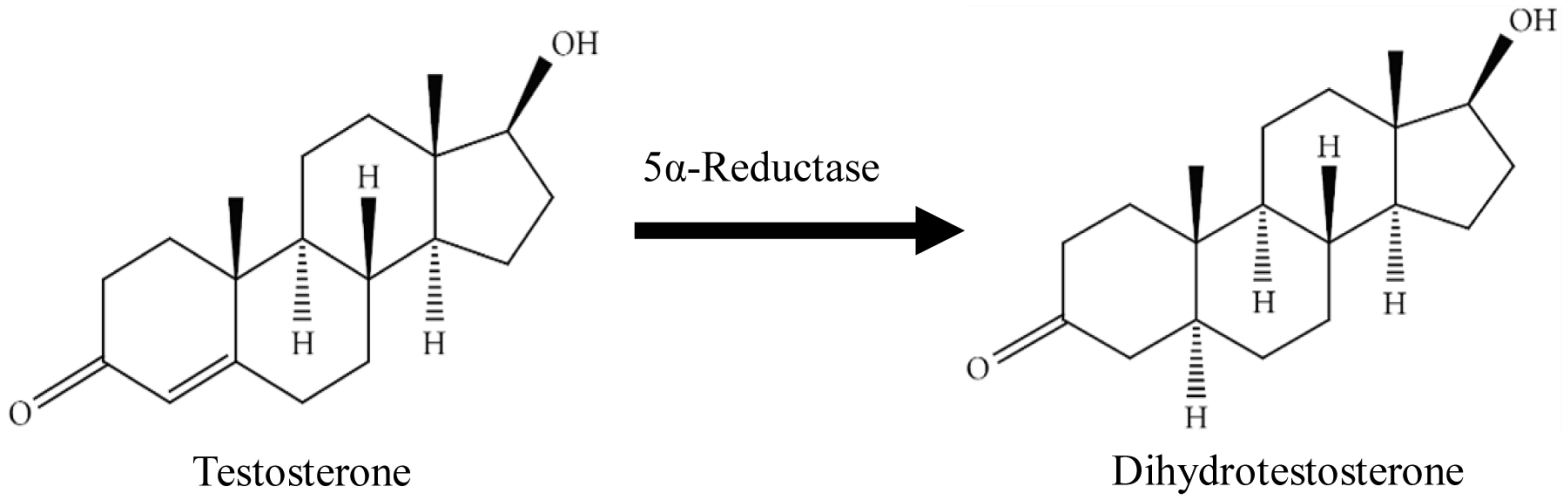
The conversion of dihydrotestosterone from testosterone by 5-alpha-Reductase. The figure is adapted and modified from National Center for Biotechnology Information (2020).

The physiologic functions and pathologic conditions of the prostate are regulated by numerous hormones and growth factors. For instance, androgens are essential for prostatic development and function as well as for cells’ proliferation and survival ( Banerjee *et al.*, 2018). Testosterone (T), the most abundant circulating androgen in male, is synthesised by the Leydig cells of the testes under the control of hypothalamus and anterior pituitary gland, and can further be converted to more potent form dihydrotestosterone (DHT) by the action of enzyme 5αR ( Figure 1). The microsomal enzyme 5αR mediates a rapid and irreversible T conversion to DHT within the prostate where it then binds to androgen receptor (AR) to exert its biological function ( Azzouni *et al.*, 2012). 5αR exists in two isoforms, namely type 1 (5αR1) and type 2 (5αR2), which differ in their molecular genetics, structural and biochemical properties, and tissue localisation ( Nahata, 2017). 5αR1 occurs predominantly in non-genital skin, the scalp, the sebaceous gland, the liver, the kidney and the brain, whereas 5αR2 is found extensively in the prostate, genital skin, seminal vesicles and in the dermal papilla ( Nahata, 2017; Othonos & Tomlinson, 2019; Strauss & FitzGerald, 2019). Both isozymes are expressed at a much lower level in other peripheral tissues. Due to the tissue specific expression of 5αRs, DHT concentration is much higher than T concentration in the prostate. Androgens, although necessary for the development of prostate, could also allow CaP cells to grow. They promote the growth of cancerous prostate cells by binding to and activating the AR, resulting in the expression of specific genes responsible for the proliferation of CaP cells. Augmented levels of androgens, particularly DHT, are detrimental towards CaP later in life.

### Inhibition of 5αRs using 5-alpha-Reductase inhibitors (5αRis)

Progression of cancer in prostate is typically dependent on the levels of androgens present during the initial stages of cancer growth ( Taplin *et al.*, 1995). Therefore, reducing the production of androgen provides a useful approach to androgen deprivation where it restricts the availability of T, allowing minimal conversion to DHT by 5αRs and androgen-receptor binding activity. The inhibition of 5αRs will subsequently limit the production of DHT and therefore represents a valid target for CaP risk prevention and reduction as well as treatment strategies as a whole.

**Figure 2.**
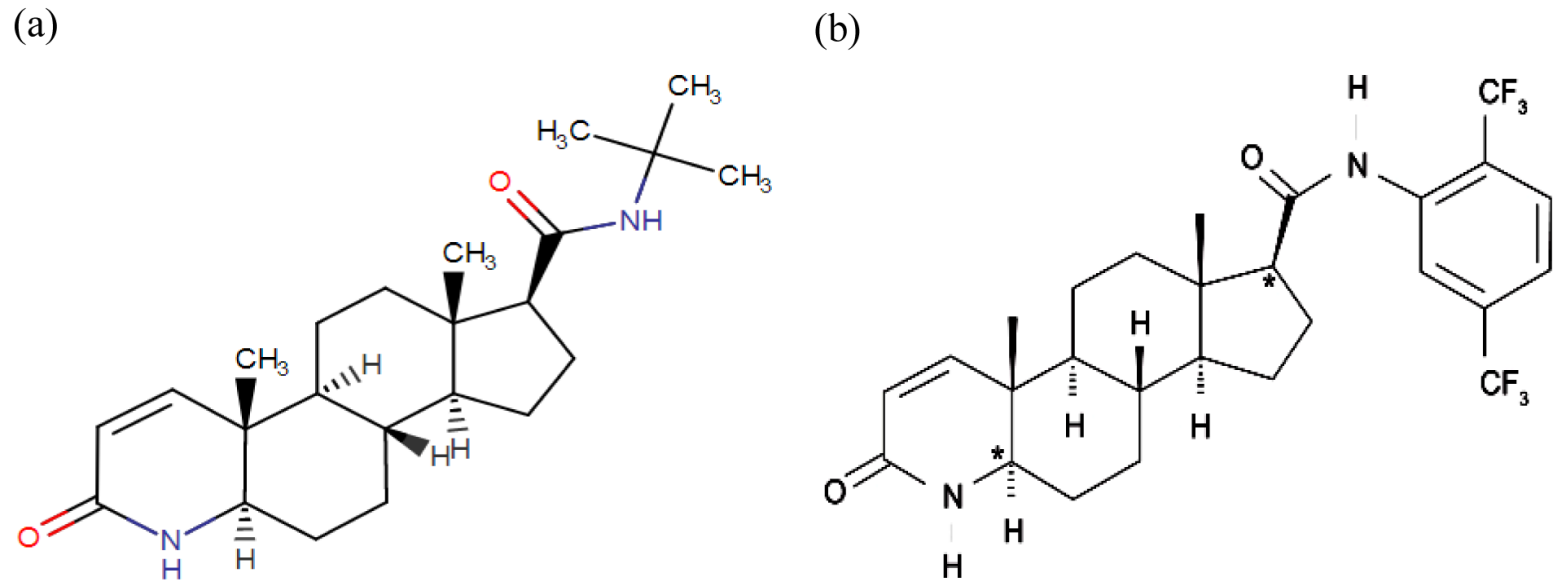
The chemical structure of 5αRis; ( **a**) Finasteride, and ( **b**) Dustasteride. The figure is adapted and modified from National Center for Biotechnology Information (2020).

Synthetic 5αR inhibitors (5αRis) can be broadly classified into two categories, namely steroidal and nonsteroidal, where their development was aimed to bind to 5αR with little or no affinity for the androgen or other steroid receptors. The most promising and well-studied 5αRis by far are finasteride and dutasteride. Clinical treatment with finasteride and dutasteride have shown to decrease both mean serum and intraprostatic level of DHT in CaP patients ( Andriole *et al.*, 2004; Clark *et al.*, 2004; McConnell *et al.*, 1992; Span *et al.*, 1999). Finasteride is the first synthetic steroidal 5αRi approved for the treatment of benign prostatic hyperplasia (BPH) and male pattern baldness ( Aggarwal *et al.*, 2010; Brough & Torgerson, 2017). Finasteride, a synthetic 4-azasteroid compound, is a potent competitive inhibitor of 5αR2 that also inhibits 5αR1 but less effectively ( Figure 2a). Finasteride has been reported to decrease LNCaP cell growth rate *in vitro* in a dose dependent manner ( Bologna *et al.*, 1995). Meanwhile, dutasteride, also a synthetic 4-azasteroid compound and an approved drug for BPH treatment, is known as a dual 5αRi with a 45-fold more effective in inhibiting 5αR1 and 2-fold more effective in inhibiting 5αR2 than finasteride ( Figure 2b). Dutasteride has been reported to inhibit T and DHT-induced LNCaP cell proliferation by targeting the 5aRs activity and displaying a more potent DHT inhibition than finasteride ( Lazier *et al.*, 2004). Dual inhibition of 5aRs is more beneficial than selective type 2 inhibition as it suppresses the DHT level to a great extent by also preventing the type 1 mediated synthesis of DHT production.

These observations, among others, provide a strong rationale for CaP risk reduction and prevention using 5αRis finasteride and dutasteride, although their use as a targeting therapeutic drug continues to be widely discussed. One of the main issues that halt the progression of 5αRis, considered as an effective CaP therapeutic agent, is the numerous undesirable side effects including erectile dysfunction, ejaculatory dysfunction and loss of libido ( Erdemir *et al.*, 2008). 5αRis, which are also commonly prescribed for women with hair loss, demonstrate headache, gastrointestinal discomfort and decreased libido as the most common reported side effects ( Hirshburg *et al.*, 2016). Other factors include the controversy that 5αRis appear to only preferentially prevent low-grade cancers and now concern lingers that 5αRis may induce or selectively promote growth of high-grade disease ( Hamilton & Freedland, 2011).

### Plants as an alternative to conventional 5αRis

Synthetic drugs are known to have various adverse effects, hence, safer alternative drugs have been sought, focusing on herbal sources. Older people often use traditional plants as complementary and/or alternative remedies to sustain healthy life or cure diseases. Traditional plants are known to be in medicinal practices for treatment of various diseases since ancient times ( Falodun, 2010; Leroi-Gourhan, 1975; Pan *et al.*, 2014) and the use of medicinal plants in the search of new drugs from nature has increased since then ( Savithramma *et al.*, 2011). Plants contain numerous bioactive compounds for treatment of many conditions, including cancer ( Mohan *et al.*, 2011). The plant kingdom is comprised of approximately 250,000 plant species and only around 10% have been studied for the treatment of different diseases ( Iqbal *et al.*, 2017). Approximately 25% of the modern drugs in clinical use are derived from plants, where the majority of these drugs were discovered as a direct result of studies focusing on the isolation of active compounds from traditional plants ( Calixto, 2019).

Herbal drugs, which have been increasingly used in cancer treatment, represent a rich pool of new, and interesting bioactive entities for the development of CaP therapeutic agents. This is because herbal plants exhibit favourable side effects and toxicity profiles compared to conventional chemotherapeutic agents. Therefore, the aim of this review is to discuss the use of phytochemicals found in various plants that have been proven to exhibit anti-CaP as alternative herbal CaP medicines and to focus on the types of phytochemical present in plants that exhibit inhibitory activities on 5αRs isozymes.

### 5αR inhibition activity by phytochemicals

Phytochemicals are the bioactive non-nutrient plant compounds that are found present in fruits, vegetables, grains and other plant foods, where its consumption has been linked to reduction on risk of many major chronic diseases ( Sathishkumar & Baskar, 2014). Six major phytochemical categories that have been identified are phenolics, alkaloids, nitrogen-containing compounds, organosulfur compounds, phytosterols and carotenoids ( Liu, 2013). The surge of interest in finding new natural bioactive entities as a template for new drug discovery and/or studying existing bioactive compounds for other biological and medicinal properties has kept scientists constantly conducting more chemical studies, particularly focusing on fractionating, isolating and identifying the active compounds. Phytochemicals offer a promising array of entities that can be further formulated into complementary or alternatives to conventional medicines that are less costly and have no/less harmful side effects. Many *in vivo* and *in vitro* studies have shown anti-CaP properties of various phytochemicals via numerous pathways as well as their ability to inhibit 5αR activity, particularly the phytosterols and phenolics, probably due to their structural similarity with the current inhibitors of 5αRs. Fatty acids, which differ in structure to any 5αRis, are also found to exhibit anti-5αR activity. Table 1 and Table 2 summarise the findings of anti-CaP studies and the inhibitory action on 5αRs of various phytochemicals, respectively.

**Table 1.** Summary of findings from anti-CaP studies of various phytochemicals.

Phytochemicals	Mechanism of action investigated/involved	CaP cells type and/or mouse model	Findings	References
β-sitosterol	Growth and metastasis of tumour cells	PC-3 in SCID mice	Slow down the tumour growth and metastasis	( Awad *et al.,* 2001)
Stigmasterol	Effect of androgen activity	LNCaP	Suppress androgen activity of DHT	( Kamei *et al.,* 2018)
Lupeol	Growth of CaP cell xenograft tumour	LAPC4, LNCaP, CRPC, CWR22Rv1 in nude mice	Effectively halt the tumour growth	( Saleem *et al.,* 2005)
Quercetin	Growth of CaP cell xenograft tumour; angiogenesis; apoptosis; proliferation	PC-3 in nude mice, LAPC-4 in SCID mice, CWR22 in SCID mice, DU-145 in nude mice	Halt the tumour growth at selective dose; Inhibit angiogenesis; induce apoptosis; inhibit proliferation	( Yang *et al.,* 2015)
Myricetin	Tumour metastasis; apoptosis	PC-3 and DU-145 in thymic nude mice	Inhibit tumour cells migration and invasion; promote cell apoptosis	( Ye *et al.,* 2018)
Fisetin	Tumour cell apoptosis; cytotoxicity; viability	PC-3, LNCaP, DU-145, CWR22Rupsilon1	Activate tumour cell apoptosis; enhance cytotoxicity; decrease tumour cell viability	( Szliszka *et al.,* 2011)
Kaempferol	CaP cell proliferation	AT6.3	Inhibit cell proliferation of in dose-dependent manner	( Wang *et al.,* 2003)
Biochanin A	Growth of CaP cell; cytotoxicity; apoptosis	LNCaP, DU-145, AT6.3	Inhibit growth of cells; augment selective-cancer cell cytotoxicity; induce cells apoptotic effects	( Wang *et al.,* 2003); ( Szliszka *et al.,* 2013)
Genistein	Growth of CaP cell; apoptosis; proliferation	LNCaP, DU-145, AT6.3	Inhibit growth of cells; inhibit cell proliferation of in dose-dependent manner	( Wang *et al.,* 2003)
Epigallocatechin-gallate	Growth of induced-CaP cell tumour	PC-3 and LNCaP in mice	Inhibit the growth and reduce the size of tumour	( Liao *et al.,* 1995)
Epicatehin-gallate	Cell viability; proliferation, apoptosis	LNCaP, PC-3	Inhibit the CaP cells viability in dose- dependent manner; inhibit cell proliferation; induce cells apoptotic effects	( Stadlbauer *et al*., 2018)
Oleic Acid	Cell proliferation	LNCaP	Inhibit cell proliferation	( Liu *et al*., 2009)
Linoleic Acid	Cell proliferation; viability	LNCaP, PC-3	Inhibit cell proliferation and viability	( Liu *et al*., 2009); ( Eser *et al*., 2013)
Myristic Acid	Growth of prostate; DHT level in prostate	Prostate in Sprague- Dawley rats	Reduce prostate growth; inhibit prostate enlargement; reduce prostate weight; reduce DHT level	( Patil & Yadav, 2016)
Lauric Acid	Growth of prostate; DHT level in prostate, cell proliferation	Prostate in Sprague- Dawley rats; LNCaP	Reduce prostate growth; inhibit prostate enlargement; reduce prostate weight; reduce DHT level; inhibit cell proliferation	( Liu *et al*., 2009); ( Patil & Yadav, 2016)

**Table 2.** Inhibitory action of various phytochemicals on 5αRs.

Phytochemical	Structures	Effect on 5αRs	IC50 (µM)	Model of Study	Type of CaP cell line studied	Source of Plant	References
**Phytosterols;**							
**β-sitosterol**	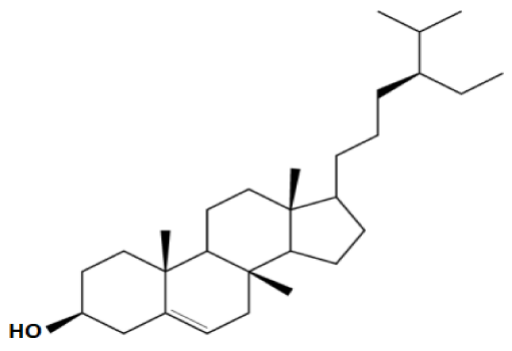	Inhibition on type I and II	2.7	*In vitro*	PC-3	*Sepenoa repen, Hypoxis* *rooperi, Secale cereale (Rye* *Grass Pollen), Urtica dioica,* *Prunus africana*	( Awad *et al*., 2001), ( Cabezal *et al*., 2003), ( Madersbacher *et al*., 2007), ( Pais, 2010), ( Dawid-Pać *et al*., 2014), ( Komakech *et al*., 2017)
**Stigmasterol**	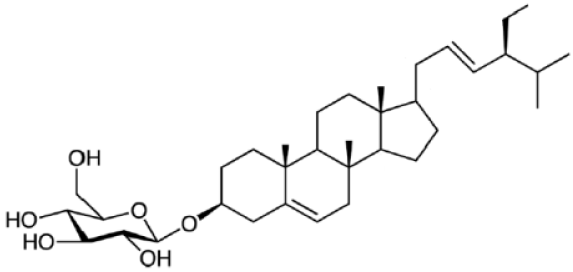	Inhibition on type I and II	27.2	*In vitro*	LNCaP	*Sepenoa repen, Phyllanthus* *urinaria, Croton sublyratus,* * Ficus hirta, Eclipta alba (L.)* *Hassk, Eclipta prostrate,* * Parkia speciosa, Gypsophila* *oldhamiana, Eucalyptus* *globules, Aralia cordata,* *Emilia sonchifolia, Akebia* *quinata, Desmodium* *styracifolium, Heracleum* *rapula*	( Iehlé *et al*., 1995), ( Pais, 2010), ( Dawid-Pać *et al*., 2014), ( Kamei *et al*., 2018)
**Lupeol**	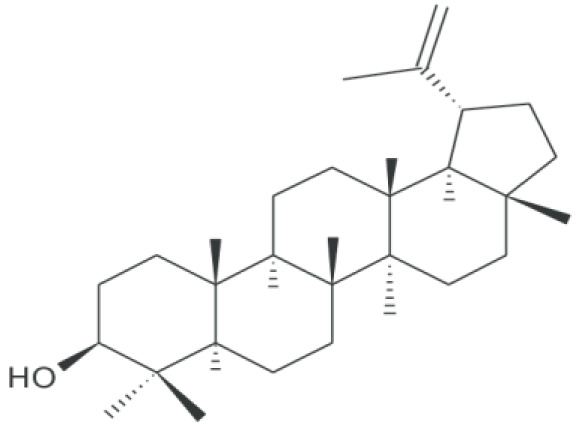	Inhibition on type I and II	15.9, 17.3 19.1 25	*In vitro*	LAPC4, LNCaP, C4-2b, 22Rv1	*Sepenoa repen,* American *ginseng,* *Shea butter plant,* *Tamarindus indica,* *Allanblackia monticola,* *Himatanthus sucuuba,* *Celastrus paniculatus,* * Zanthoxylum riedelianum,* *Leptadenia hastata, Crataeva* * nurvala, Bombax ceiba,* *Sebastiania adenophora*	( Siddique *et al.*, 2011) ( Rainer *et al*., 2007)
**Phenolics (Polyphenols);**							
**Quercetin**	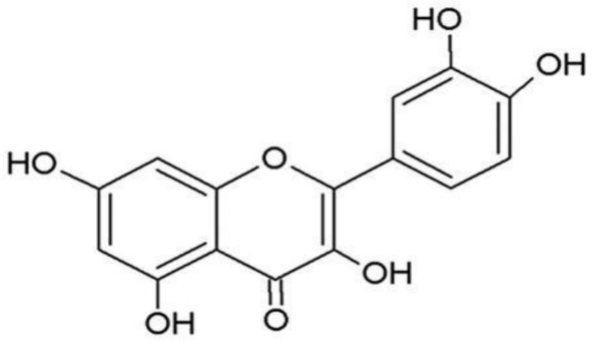	Inhibition on type I	23	*In vitro*	PC-3, LNCaP, DU-145	*Morus alba L, Camellia* *chinensis, Allium fistulosum,* * Calamus scipionum, Moringa* *oleifera, Centella asiatica,* *Hypericum hircinum,* *Hypericum perforatum*	( Hiipakka *et al*., 2002), ( Salvamani *et al*., 2014), ( Yang *et al*., 2015), ( Kashyap *et al*., 2019)
**Myricetin**	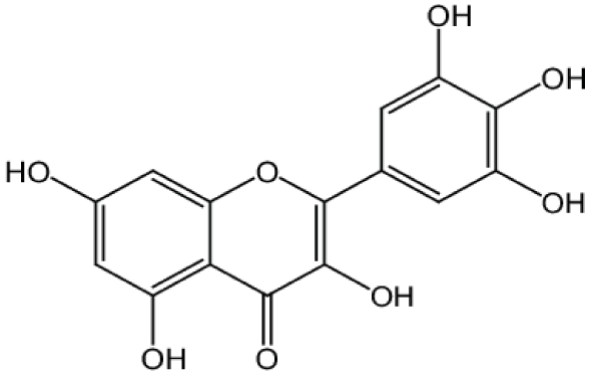	Inhibition on type I	23	*In vitro*	PC-3, DU-145	*Ampelopsis cantoniensis,* *Myrica cerifera L, Calamus* * scipionum, Chrysobalanus* *icaco L, Moringa oleifera, Aloe* * vera*	( Hiipakka *et al*., 2002), ( Salvamani *et al*., 2014) ( Ye *et al*., 2018)
**Fisetin**	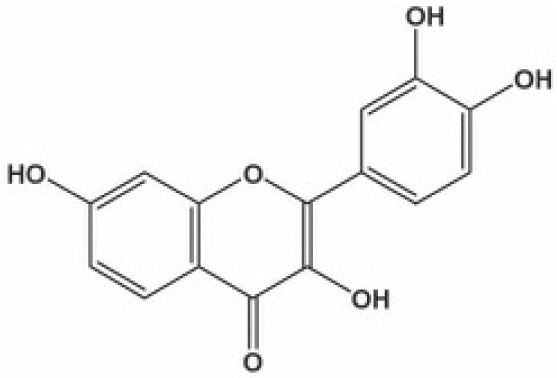	Inhibition on type I	57	*In vitro*	PC-3, LNCaP, DU-145, CWR22 Rupsilon1	*Butea frondosa, Gleditsia* *triacanthos, Quebracho * *colorado, Curcuma longa,* *Rhus verniciflua, Acacia * *greggii, Acacia berlandieri*	( Hiipakka *et al*., 2002), ( Khan *et al*., 2008), ( Szliszka *et al*., 2011) ( Salvamani *et al*., 2014) ( Kashyap *et al*., 2019)
**Kaempferol**	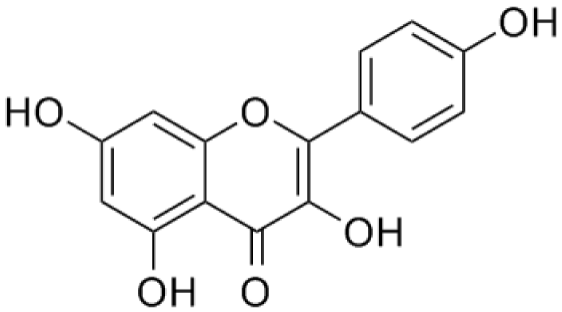	Inhibition on type II	12	*In vitro*	AT6.3 rat	*Moringa oleifera, Centella* *asiatica, Euonymus alatus,* * Kaempferia galanga L,* *Ginkgo biloba, Equisetum* *spp., Tilia spp., Sophora* *japonica, propolis*	( Hiipakka *et al*., 2002), ( Wang *et al*., 2003) ( Park *et al.*, 2006)
**Biochanin A**	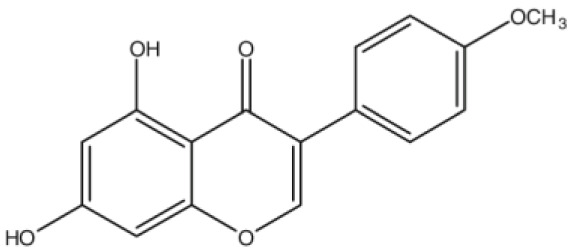	Inhibition on type II	17	*In vitro*	LNCaP, DU-145, AT6.3 rat	*Trifolium pratense L, Glycine* *max, Lupinus*	( Peterson & Barnes, 1993), ( Wang *et al*., 2003), ( Jlan, 2009), ( Szliszka *et al*., 2013) ( Spagnuolo *et al*., 2015), ( Zhang *et al*., 2016)
**Genistein**	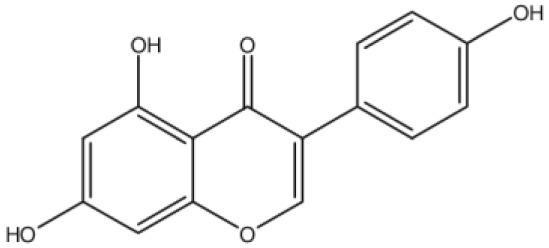	Inhibition on type II	23	*In vitro*	LNCaP, DU-145, AT6.3 rat	*Glycine max, Lupinus*	( Peterson & Barnes, 1993), ( Wang *et al*., 2003), ( Jlan, 2009), ( Spagnuolo *et al*., 2015), ( Zhang *et al*., 2016)
**Daidzein**	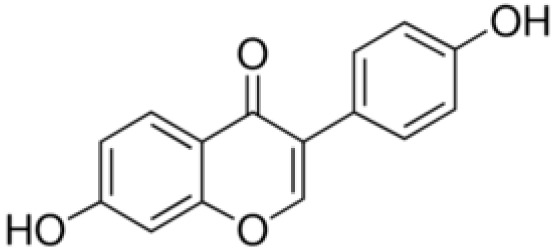	Inhibition on type II	29	*In vitro*	LNCaP, DU-145, AT6.3 rat	*Glycine max, Lupinus*	( Peterson & Barnes, 1993), ( Wang *et al*., 2003) ( Jlan, 2009), ( Spagnuolo *et al*., 2015), ( Zhang *et al*., 2016)
**Phenolics (Catechins);**							
**Epicatechin-gallate**	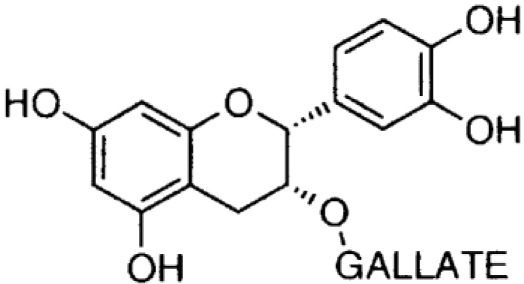	Inhibition on type I	11	*In vitro*	PC-3, LNCaP, DU-145	*Camella sinensis, Betula* *pubescens, Betula pendula,* * Cocos nucifera,* fruit pulp of * Argania spinosa, Cassia* * fistula*	( Agarwal, 2000), ( Chung *et al*., 2001), ( Hiipakka *et al*., 2002) ( Stadlbauer *et al*., 2018)
**Epigallocatechin-** **gallate**	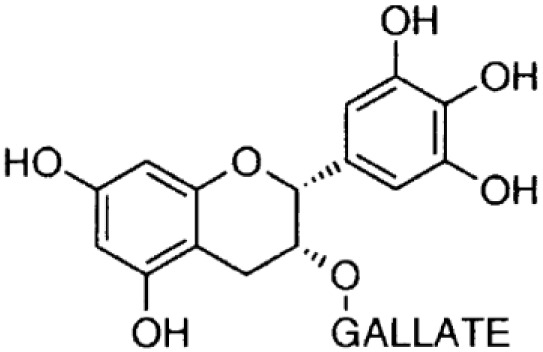	Inhibition on type I	15	*In vitro*	PC-3, LNCaP, DU-145	*Camella sinensis, Betula pubescens, Betula pendula, * *Cocos nucifera,* fruit pulp of * Argania spinosa, Cassia* * fistula*	( Agarwal, 2000), ( Chung *et al*., 2001), ( Hiipakka *et al*., 2002)
**Fatty Acids;**							
**Oleic Acid**	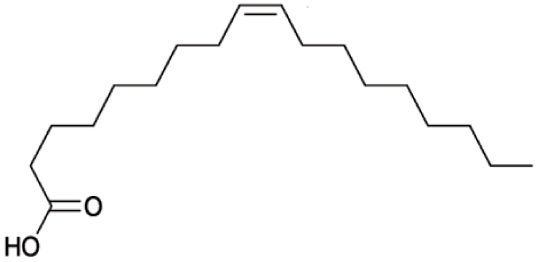	Inhibition on type I	14.2	*In vitro*	LNCaP	*Sepenoa repens, Helianthus* *annuus*	( Raynaud *et al*., 2002), ( Liu *et al*., 2009), ( Sheeba *et al*., 2015)
**Linoleic Acid**	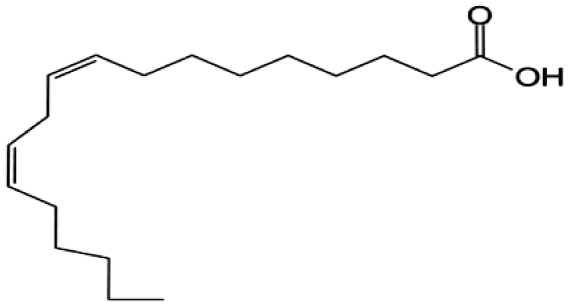	Inhibition on type I	46.4	*In vitro*	LNCaP, PC-3	*Sepenoa repens, Prunus* *africana,* *Cocos nucifera, Helianthus* *annuus*	( Raynaud *et al*., 2002), ( de Lourdes Arruzazabala *et al*., 2007), ( Liu *et al*., 2009), ( Eser *et al*., 2013), ( Nyamai *et al*., 2015), ( Sheeba *et al*., 2015)
**Myristic Acid**	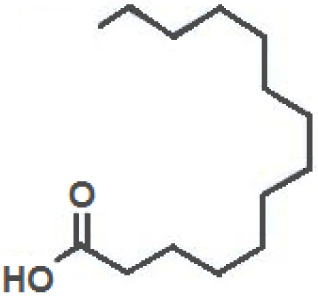	Inhibition on type II	18.8	*In vitro*	Cell expression system in Sf9	*Sepenoa repens*, *Prunus* *africana*	( Raynaud *et al*., 2002)
**Lauric Acid**	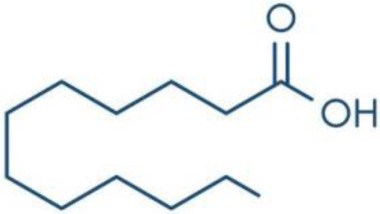	Inhibition on type I and II	92.8	*In vitro*	LNCaP	*Sepenoa repens*, *Prunus* *africana,* *Cocos nucifera*	( Raynaud *et al*., 2002), ( de Lourdes Arruzazabala *et al.*, 2007),( Liu *et al*., 2009) ( Nyamai *et al.*, 2015) ( Komakech *et al*., 2017)

***1. Phytosterols***

Plant sterols or phytosterols (PS) are bioactive components in plants with 28- or 29-carbon alcohols and double bonds at the C-5 position of the ring that resemble cholesterol in vertebrates in terms of both of their structure and function ( Zaloga, 2015). More than 200 different types of phytosterols have been reported, with β-sitosterol, campesterol and stigmasterol being the most abundant type of PS ( Miras-Moreno *et al.*, 2016). The toxicity profiles of PS have shown that there are no obvious side effects after long-term feeding of PS in both animals and humans ( Ling & Jones, 1995). PS play essential roles in the reduction of cholesterol in blood that eventually decrease cardiovascular morbidity, therefore are well known for their beneficial effect on cardiovascular disease risk. Katan *et al.* (2003) reported that the intake of 1–2 g of PS daily can effectively lower low-density lipoprotein cholesterol levels by 8%-12%. However, little attention was received with regard to PS on their potential in cancer aetiology, although increasing evidence of biochemical and molecular effects of PS may make them strong candidates for cancer therapeutic agents.

Being structurally similar with four rings to synthetic 5αRis finasteride and dutasteride, PS could stand as the strongest promising candidate for plant-derived 5αRis. A study by Awad *et al.* (2001) showed that β-sitosterol inhibits the growth and migration of PC-3 human CaP and slows down the growth of prostate tumour in SCID mice, which suggests an involvement of androgenic mechanism of action as CaP is dependent on androgen. An *in vitro* metabolic study in hamster prostate by Marisa Cabeza and colleagues revealed that β-sitosterol inhibits the enzymatic activity of 5αRs in dose-dependent manner, which therefore confirms the ability of β-sitosterol as a 5αRi ( Cabezal *et al.*, 2003).

Another PS, stigmasterol, was reported to be associated with a reduction in common cancer risks including colon cancer, breast cancer and CaP ( Bradford & Awad, 2007). Kamei *et al.* (2018) studied *Phyllanthus urinaria* where the extract was shown to suppress androgen activity of DHT in LNCaP cell lines and has inhibitory activity against 5αRs, of which the active bioactive compound responsible for the activity was identified as stigmasterol isolated from an activity-guided fractionation. An *in vitro* study of *Serenoa repens* extract (SPE) using baculovirus-directed insect cell expression system demonstrated the inhibition of both 5αR1 and 5αR2 in a non-competitive and uncompetitive manner, respectively ( Iehlé *et al.*, 1995). The major active compounds from PS of SPE includes β-sitosterol and stigmasterol ( Suzuki *et al.*, 2009). SPE, a well-known phytotherapeutic agent, most frequently used to treat lower urinary tract symptoms and as a BPH medicine, not only targets the regulation of 5αRs activity but also hampers the binding of DHT to androgenic receptors ( Dawid-Pać *et al.*, 2014). Pais (2010) reported in his study that in a cell-free test system, ethanolic extract of *Serenoa repens* was a potent inhibitor of 5αR2 with 61% inhibition. From these observations, β-sitosterol and stigmasterol are found to exhibit inhibitory activity on both isozymes of 5αRs. Various plants reported to have β-sitosterol as their major active compound include *Hypoxis rooperi* extract (Harzol®), *Secale cereal* (Rye Grass Pollen), *Urtica dioica* and *Prunus africana* ( Komakech *et al.*, 2017; Madersbacher *et al.*, 2007). A study by Nahata & Dixit (2014) analysing the inhibitory effects of different types of *Urtica dioica* extracts on the activity of 5αR2, demonstrated that ethanolic extracts were the best 5αRis, followed by petroleum ether and aqueous extracts. Stigmasterol, with known 5αR2 inhibitory activity, is also reported to be present in various medicinal plants including *Croton sublyratus, Ficus hirta, Eclipta alba (L.) Hassk, Eclipta prostrate, Parkia speciosa, Gypsophila oldhamiana, Eucalyptus globules, Aralia cordata, Emilia sonchifolia, Akebia quinata, Desmodium styracifolium, Heracleum rapula* ( Chaudhary *et al.*, 2011).

Lupeol, another PS, has also been shown to exhibit various pharmacological properties including anti-CaP activity ( Siddique & Saleem, 2011). Siddique *et al.* (2011) demonstrated in their study that lupeol inhibited the growth of various CaP cells i.e LAPC4, LNCaP and CRPC cells, *in vitro*. Another *in vivo* study using implanted CaP cells as xenograft tumours in mice also revealed that lupeol treatment effectively halts tumour growth, which further suggests the ability of lupeol as an effective agent that can potentially inhibit the tumourigenenicity of CaP cells. Lupeol has also been observed to have a striking ability to preferentially kill CaP cells while sparing normal prostate epithelial cells ( Saleem *et al.*, 2005). SPE, which contains lupeol as its bioactive compound, has been shown to possess a dual 5αRs inhibition activity ( Iehlé *et al.*, 1995; Rainer *et al.*, 2007), therefore confirming the ability of lupeol to inhibit both 5αR1 and 5αR2. Lupeol can also be found in other numerous medicinal plants such as American ginseng *,* Shea butter plant *, Tamarindus indica, Allanblackia monticola, Himatanthus sucuuba, Celastrus paniculatus, Zanthoxylum riedelianum, Leptadenia hastata, Crataeva nurvala, Bombax* ceiba and *Sebastiania adenophora* ( Siddique & Saleem, 2011) *.* PS, being able to exhibit dual inhibition on both isoforms of 5αRs, further strengthens its potential as the most promising candidate as plant-derived 5αRis.

***2. Phenolics***

***a) Polyphenols***

Polyphenols (PP) are generally subdivided into two large groups: flavonoids and non-flavonoids. For centuries, preparation containing PP-flavonoids were applied as major active components in different remedies which were used to treat different human diseases ( Salvamani *et al.*, 2014). PP exert various pharmacological effects such as anti-oxidant, anti-hypertensive, anti-inflammatory and anti-thrombotic activity that can further help in promoting human health ( Hollman *et al.*, 1997; Kleemann *et al.*, 2011; Manach *et al.*, 2005; Vinson *et al.*, 1995). The toxicity profiles have shown that PP exert their therapeutic effect in a dosage-dependent manner in animal studies, whereas moderate dosages of PP do not seem to elicit any adverse effects, hence indicating its beneficial effects and safe use. Conversely, at high dosages, PP might show parallel adverse effects and/or toxicity, particularly due to accumulation of high levels of PP ( Silva & Pogačnik, 2020).

PP, although lacking one ‘ring’, exhibit a chemical structure similar to the synthetic 5αRis, hence representing a potential plant-derived 5αRis candidate. Quercetin, one of the PP-flavonoids, has a 3-OH group on its pyrone ring and is abundant in many fruits and vegetables. It has been shown to be non-toxic and possesses an anti-cancer property in various human cancer cell lines both *in vitro* and *in vivo* including CaP ( Piao *et al.*, 2014). *In vitro*, quercetin exhibits significant arrest of cell cycle, decreases cell viability, inhibits proliferation, and induces cell apoptosis especially in PC-3, LNCaP and DU-145 cell lines, whereas when used *in vivo*, growth of a CaP cell xenograft tumour was effectively halted at a selective dosage ( Yang *et al.*, 2015). Another PP, myricetin, possesses an aglycone structure that has been thought to attribute strongest inhibitory effects on enzymes such as DNA polymerases and DNA topoisomerase II and hence interferes with cellular proliferation activities ( Shiomi *et al.*, 2013). Myricetin has been reported to exhibit anti-tumour activity in *in vitro* (DU-145 and PC-3 cell lines) and *in vivo* (thymic nude mice) models, by promoting cell apoptosis and inhibition of cell migration and invasion ( Ye *et al.*, 2018).

Another PP, fisetin, which has two aromatic rings linked via a 3-C oxygenated heterocyclic ring with four hydroxyl groups and one oxo group, has also shown remarkable anti-cancer effects in multiple *in vitro* and *in vivo* systems. Fisetin-promoted apoptotic activation was seen in DU-145, LNCaP, and PC-3 human CaP cells ( Szliszka *et al.*, 2011). Khan & colleagues (2008) conducted a study to determine whether fisetin inhibits cell growth and induce apoptosis in human CaP cells, where the study revealed fisetin treatment decrease the viability of LNCaP, CWR22Rupsilon1 and PC-3 cells while exerting only minimal effects on normal prostate epithelial cells. Fisetin arrested the G _1_-phase cell cycle activity in LnCAP cells and induced cell apoptosis ( Khan *et al.*, 2008). A study by Szliszka *et al.* (2011) has also demonstrated fisetin’s ability to enhance cytotoxicity and apoptosis in LNCaP, DU-145 and PC-3 cells. From all of the outcomes, the PP quercetin, myricetin, and fisetin present a significant role and impact towards CaP treatment strategies via numerous pathways and this includes targeting the inhibition of 5αRs activity. An extensive study conducted by Hiipakka *et al.* (2002) to determine inhibition of 5αRs using varieties of polyphenols in cell-free assay and whole-cell assay, showed that PP quercetin, myricetin and fisetin were more potent against 5αR1 than 5αR2 isozyme (IC _50_ < 100 µM) in cell-free assay but showed little or no activity in whole-cell assay. Structure-activity relationships were also examined where it appeared that the number and position of B-ring hydroxyl groups were important for inhibitory activity against 5αR1. Many plants are reported to contain PP like quercetin, myricetin and fisetin. For example, *Camellia chinensis, Allium fistulosum, Calamus scipionum, Moringa oleifera, Centella asiatica, Hypericum hircinum* and *Hypericum perforatum* have been reported to have high contents of quercetin ( Salvamani *et al.*, 2014). High contents of myricetin has also been reported in *Myrica cerifera L, Calamus scipionum, Chrysobalanus icaco L, Moringa oleifera and Aloe vera* ( Salvamani *et al.*, 2014) *.* While plants like *Butea frondosa, Gleditsia triacanthos, Quebracho colorado, Curcuma longa, Rhus verniciflua, Acacia greggii* and *Acacia berlandieri* are rich sources of fisetin ( Salvamani *et al.*, 2014). 

Several other PP have also exhibited anti-CaP effects. The effect of the PP, genistein, daidzein, and biochanin A on the growth of LNCaP and DU-145 human CaP cell lines was studied where all except daidzein inhibited the cells growth ( Peterson & Barnes, 1993). Wang & colleagues (2003) studied the PP reduction effect on CaP cell proliferation and apoptotic resistance *in vitro* using a AT6.3 rat CaP cell line and revealed that the PP kaempferol, biochanin A, and genistein were responsible for inhibited cell proliferation in a dose-dependent manner and induced apoptotic effects, except for daidzein, which counteracted the effect ( Wang *et al.*, 2003). Szliszka *et al.* (2013) in their study demonstrated that biochanin A remarkably augmented selective-cancer cell cytotoxicity and apoptosis in both LNCaP and DU-145 cell lines. Many *in vivo* and *in vitro* studies have demonstrated PP’s ability as 5αRis in combating CaP ( Evans *et al.*, 1995; Hiipakka *et al.*, 2002; Park *et al.*, 2003). Kaempferol, biochanin A and genistein were found to be more effective as inhibitors of 5αR2 than 5αR1 in a cell-free assay as well as significantly inhibit 5αR2 in a whole-cell assay ( Hiipakka *et al.*, 2002). A previous study has also demonstrated genistein and biochanin A as potent inhibitors of 5αRs, more specifically on type 2 in human genital skin fibroblasts and BPH tissue homogenates and on type 1 in prostate tissue homogenates ( Evans *et al.*, 1995). A study that used isolated kaempferol from *Camellia sinensis* showed good inhibition on 5αR2 in HEK-293 cells lines that expressed both 5αRs type 1 and 2 ( Park *et al.*, 2006). Park *et al.* (2003) revealed that *Thujae occidentalis semen* (TOS) extract showed high inhibition activity on 5αR2 that were expressed in HEK-293 cell lines. Previous studies have shown that TOS extracts contain PP flavonoids, which suggests a promising potential of PP as strong inhibitors of 5αRs ( Hidehiko *et al.*, 1996). Kaempferol has been identified in many other plants including *Centella asiatica, Euonymus alatus, Kaempferia galanga L, Ginkgo biloba, Equisetum spp., Tilia spp., Sophora japonica* and *propolis* ( Salvamani *et al.*, 2014). Genistein, daidzein and biochanin A which are the isoflavones that are mostly found in soybean ( *Glycine max*), lupin *(Lupinus)* and red clover ( *Trifolium pratense L*).

***b) Catechin***

Catechin is a type of PP that is found abundant especially in green tea. Two out of four major types of catechin are discussed herein, namely epigallocatechin-gallate (EGCG) and epicatechin-gallate (ECG). An *in vivo* study where PC-3 and LNCaP cell lines from tumour-induced mice was injected with EGCG revealed that within seven days the EGCG rapidly inhibited the growth and reduced the size of the CaP tumours ( Liao *et al.*, 1995). Kao *et al.* (2000) found that EGCG reduces blood levels of T as well as prostate growth. Stadlbauer *et al*. (2018) studied the anti-tumour effect of ECG *in vitro* and demonstrated that the treatment of LNCaP and PC-3 cell lines using ECG inhibited cell viability in a dose-dependent manner. Both EGCG and ECG were also reported to have significant inhibitory effects on cell proliferation and induced apoptosis in DU-145 cells ( Agarwal, 2000; Chung *et al.*, 2001). In regard to catechin as a 5αRi, a previous study using rat liver microsomes that expressed different types of 5αRs via retroviral expression vector pMV7 system has shown that ECG and EGCG are potent inhibitors of 5αR1 but not of 5αR2 ( Liao & Hiipakka, 1995). A further extensive 5αRis study by Hiipakka *et al.* (2002) using a similar method as previous has demonstrated that ECG and EGCG were better inhibitors against 5αR1 than 5αR2. An *in vitro* study by Koseki *et al.* (2015) showed the reduction in DHT conversion from T in 5αRs enzymatic activity in rat liver microsomes using Quercus acutissima extract where both EGCG and ECG were identified as being amongst the major components in the extract. Catechins are found in other plants such as *Betula pubescens, Betula pendula, Cocos nucifera,* fruit pulp of *Argania spinosa* and *Cassia fistula* ( Hiipakka *et al.*, 2002).

***3. Fatty acids***

Fatty acids (FA) are monocarboxylic acids containing long hydrocarbon chains found naturally in various plants and in general can either be saturated or unsaturated ( Jóźwiak *et al.*, 2020). Saturated FA includes myristic acid (MA) and lauric acid (LA), which are a long-chain fatty acid with a 14-carbon backbone and medium-chain fatty acid with a 12-cabon backbone, respectively. Oleic acid (OA) and linoleic acid (LNA) are mono-unsaturated omega-9 FA and poly-unsaturated omega-6 FA, respectively. Toxicity profiles of FA demonstrate positive impacts on various tissues as they generally pose no significant safety concern but have only low systemic toxicity potential ( Burnett *et al.*, 2017; Karacor & Cam, 2015).

There are various studies that showed a decreased incidence of CaP with consumption of a FA-rich diet, especially from marine-derived FA, although knowledge on the effect of plant-derived FA on CaP remains limited. A clinical study that aimed to investigate the association of FA with risk of CaP in a case-control study of 209 CaP patients and 224 cancer-free men revealed that FA reduced the risk of CaP ( Jackson *et al.*, 2012). In an *in vivo* study by de Lourdes Arruzazabala *et al.* (2007) that determined the effect of coconut oil (CO), which is rich in MA and LA, on uncontrolled growth of prostate gland using Sprague-Dawley rats, it was found that CO significantly reduced the prostate growth, suggesting that CO MA/LA-rich content could be attributed to the outcomes. This is further supported by a 14-day study by Babu *et al.* (2010) that showed MA/LA treatment in rats significantly inhibited prostate enlargement, and a four-week study by Patil & Yadav (2016) where treatment with MA and LA in rats led to significant reduction in prostate weight and DHT level in prostate.

An *in vitro* study showed that LA, OA and LNA showed proliferation inhibitory effect on LNCaP cell lines ( Liu *et al.*, 2009). Another study also demonstrated LNA effects on CaP cell proliferation where it inhibited cell viability in PC-3 and LNCaP cell lines ( Eser *et al.*, 2013). *Prunus africana* bark extracts, where amongst the major compounds identified are MA, LA and LNA, exhibit a very strong anti-androgenic activity and can prevent proliferation and kill CaP tumour cells ( Nyamai *et al.*, 2015). Oils of *Cocos nucifera* and *Helianthus annuus* contains unsaturated FA, OA, and LNA as their major components ( de Lourdes Arruzazabala *et al.*, 2007; Sheeba *et al.,* 2015). FA therefore represent a noteworthy contribution in both prevention and treatment of CaP through animal model and cell culture studies by mediating its effect in various pathways including via the inhibition of 5αRs enzymatic activity. Raynaud *et al.* (2002) conducted an extensive study on *Serenoa repens* lipido-sterolic extracts, which are mainly constituted of FA MA, LA, OA and LNA, for its inhibitory effects on 5αR enzymatic activity. The study determined the specificity of each FA inhibitory effect on both isozymes of 5αRs that have been cloned and expressed in the baculovirus-directed insect cell expression system *Spodoptera frugiperda* (Sf9). The results showed OA and LNA to be more potent against 5αR1 than 5αR2, while LA was found to be potent against both 5αR1 and 5αR2, whereas, the inhibitory effect of MA was found only active against type 2 and therefore, is a potent inhibitor of 5αR2. 

## Discussion and conclusions

CaP is one of the leading causes of death in men worldwide ( Daniyal *et al.*, 2014). Until today, various preventive and treatment strategies have been carried out to tackle the disease ( Tindall & Rittmaster, 2008). The androgens, which are the modulator of prostate growth, are also thought to contribute to the pathogenesis of CaP. This in turn, has led to a surge of interest in studies that aim to block the activity of 5aRs using available synthetic inhibitors of 5aRs resulting in androgens deprivation as part of the strategies. The idea therefore represents a valid strategy for CaP prevention and treatment. However, the use of synthetic 5αRis such as finasteride and dutasteride as 5aR activity-targeting CaP medicines continues to be widely discussed. 5aRis have been reported to have numerous adverse side effects ( Erdemir *et al.*, 2008; Hirshburg *et al.*, 2016). Due to this, study interests have switched to finding a safer remedy with no/less harmful side effects by means of natural-derived entities found in plants as an alternative to synthetic 5αRis. Plants are constituted of numerous bioactive compounds and are proven to have various powerful medicinal properties that could contribute significantly towards a healthier life ( Mohan *et al.*, 2011; Sathishkumar & Baskar, 2014). The phytochemical PS, PP and FA are discussed in this review for their potential as CaP medicines and 5αRis. Numerous *in vitro* studies using different type of CaP cell lines and *in vivo* studies using xenograft/tumour-induced animal models have revealed the ability of PS, PP and FA as potential CaP medicines targeting various mechanisms including inhibiting cell proliferation, migration and invasion, as well as promoting selective tumour cell apoptosis. In addition, the ability of PS, PP and FA as potential naturally-derived 5αRis is also demonstrated in many studies, which further validates their exhibition of anti-5αR enzymatic activity that can produce beneficial interference in androgen-dependent CaP progression. In terms of structural similarities to current synthetic 5αRis, PS that are characterised with four ‘rings’ stand as the most promising candidate for naturally-derived 5αRis and they are found to be potent against both 5αR1 and 5αR2. PP have also demonstrated anti-5αR activity on both 5αR1 and 5αR2 despite lacking one ‘ring’. FAs that exist in either saturated or unsaturated forms do not display any structural similarities to the synthetic 5αRis, but are also reported to have significant inhibitory effect against both 5αRs. All of these observations suggest a strong implication of various phytochemicals, especially PS, PP, and FA as potential CaP medicines targeting 5αR activity. These findings are hoped to assist in the next stage of human clinical trials, as to date, only synthetic 5aRis are investigated in such setting. However, further isolation of these phytochemicals needs to be done especially from the plant sources before it can be implied in human clinical setting. In conclusion, plants represent a reservoir of novel phytochemicals that can further provide a promising line on the development of CaP therapeutic agents, especially in targeting the inhibition of 5αR enzymes.

## Data availability

No data are associated with this article.
