# Supplementary material for: Phytochemicals With Anti 5-alpha-reductase Activity: A Prospective For Prostate Cancer Treatment
Source: F1000Res. 2021 Jul 6;10:221. Originally published 2021 Mar 18. [Version 3] doi: 10.12688/f1000research.51066.3 (PMC8276191; doi:10.12688/f1000research.51066.3)
Supplement: Peer review history

# Peer review: Reviewer response for version 3

**DOI:** 10.5256/f1000research.58324.r89048

**Published:** 2021-07-12

**Authors:** Martin Read

**Affiliations:** 1 Institute of Metabolism and Systems Research, University of Birmingham, Birmingham, UK

I would like to thank the authors for addressing all of my concerns.

 There is a minor correction that needs to be addressed in Table 1: “Inhibit angiogenesis” should be “inhibit angiogenesis” under findings for Quercetin. Once this minor correction has been made then the article can be indexed.

Is the review written in accessible language?

Yes

Are all factual statements correct and adequately supported by citations?

Yes

Are the conclusions drawn appropriate in the context of the current research literature?

Yes

Is the topic of the review discussed comprehensively in the context of the current literature?

Yes

Reviewer Expertise:

Endocrine Cancer, Drug Discovery, Bioinformatics

I confirm that I have read this submission and believe that I have an appropriate level of expertise to confirm that it is of an acceptable scientific standard.

# Peer review: Reviewer response for version 2

**DOI:** 10.5256/f1000research.57107.r87449

**Published:** 2021-06-21

**Authors:** Binesh Shrestha

**Affiliations:** 1 Department of Chemical Biology and Therapeutics, Novartis Institutes For Biomedical Research, Basel, Switzerland

*"Prunus Africana" *has not been changed in-text yet and in reference as well. Please use the find command and change it.

*"Quercus acutissima"* in-text has not been changed to italics.

 Others are fine. Once the above corrections are made, the article can be indexed.

Is the review written in accessible language?

Partly

Are all factual statements correct and adequately supported by citations?

Yes

Are the conclusions drawn appropriate in the context of the current research literature?

Yes

Is the topic of the review discussed comprehensively in the context of the current literature?

Yes

Reviewer Expertise:

Protein Biochemistry, Microbiology and Biotechnology

I confirm that I have read this submission and believe that I have an appropriate level of expertise to confirm that it is of an acceptable scientific standard.

# Peer review: Reviewer response for version 1

**DOI:** 10.5256/f1000research.54175.r85610

**Published:** 2021-05-25

**Authors:** Martin Read

**Affiliations:** 1 Institute of Metabolism and Systems Research, University of Birmingham, Birmingham, UK

5-alpha-Reductase inhibitors (5αRis) used in the treatment of prostate cancer are reported to have numerous adverse side effects. Here, the authors report on plant-derived alternatives that might offer favourable side effects and less toxic profiles. In particular, the authors discuss the inhibitory activities of phytochemicals on 5αRs isozymes and their structural similarities with current 5αRis used in prostate cancer therapy. Overall, this review article is clear, follows a logical narrative, and is relatively well-written. I have a few minor suggestions:

1. There are a few minor edits below that need to be made in the abstract and introduction to improve clarity:
  Abstract: “This has led to a surge of interests on plant-derived alternatives” should be “This has led to a surge of interest on plant-derived alternatives”.
  Abstract: “Therefore, in this review, we aim to discuss on the use of phytochemicals namely phytosterols” should be “Therefore, in this review, we aim to discuss the use of phytochemicals namely phytosterols”.
  Introduction: “The latter strategy was largely anticipated, considering CaP as being hormones-driven disease” should be “The latter strategy was largely anticipated, considering CaP is a hormones-driven disease”.
  Introduction: The authors should clarify the sentence “Testosterone (T), synthesised by the Leydig cells of the testes under the control of hypothalamus and anterior pituitary gland, is the most abundant circulating androgen in males, where from it, more potent form dihydrotestosterone (DHT) is synthesised”.
2. It was unclear from Table 1 which type of phytochemical (i.e., PS, PP and FA) was the most effective at inhibiting the activity of 5αRs. Would it be possible for the authors to include some information (e.g., IC50 values or equivalent) to give an indication of the relative efficacies of the different phytochemicals in the inhibition of 5αRs (type I/II)? Information on the type of prostate cancer cell lines used in each study would also be helpful to include in Table 1.
3. The data shown in Table 1 is based on *in vitro* data. It would be useful to the readers if the authors included a second table giving an overview of how the different phytochemicals (i.e., PS, PP and FA) have been studied *in vivo* (i.e., mice models). In particular, the table should include information on the types of prostate cancer mouse models, the type of anti-cancer mechanisms investigated, outcomes, etc. The inclusion of this table would give important context on how these phytochemicals (i.e., PS, PP and FA) have been investigated *in vivo* and how much more work is needed.
4. It would be helpful if the authors provided additional context in the discussion and included a sentence on how many (if any) of the phytochemicals (i.e., PS, PP and FA) have been investigated in human clinical trials to inhibit 5αRs, and what still needs to be done in future studies.

Is the review written in accessible language?

Yes

Are all factual statements correct and adequately supported by citations?

Yes

Are the conclusions drawn appropriate in the context of the current research literature?

Yes

Is the topic of the review discussed comprehensively in the context of the current literature?

Yes

Reviewer Expertise:

Endocrine Cancer, Drug Discovery, Bioinformatics

I confirm that I have read this submission and believe that I have an appropriate level of expertise to confirm that it is of an acceptable scientific standard, however I have significant reservations, as outlined above.

# Response

**Published:** 2021-06-30

**Authors:** Sheikh Naeem Shafqat

**Affiliations:** Universiti Brunei Darussalam, Brunei

Reply to the comments made by the Reviewer:

 Thank you for reviewing and suggesting some constructive amendments to the review article. Following are the actions taken in response to the reviewer’s comments:

1. The comments made by the reviewer for the Abstract and Introduction to improve the clarity of certain phrases have now been addressed and rewritten as suggested.
   
  - Abstract: “This had led to a surge of interests on plant-derived alternatives” changed to “This had led to a surge of interest on plant-derived alternatives”.
  - Abstract: “Therefore, in this review, we aim to discuss on the use of phytochemicals namely phytosterols” changed to “Therefore, in this review, we aim to discuss the use of phytochemicals namely phytosterols”
  - Introduction: “The latter strategy was largely anticipated, considering CaP as being hormones-driven disease” changed to “The latter strategy was largely anticipated, considering CaP is a hormones-driven disease”.
  - Introduction: “Testosterone (T), synthesised by the Leydig cells of the testes under the control of hypothalamus and anterior pituitary gland, is the most abundant circulating androgen in males, where from it, more potent form dihydrotestosterone (DHT) is synthesised” changed to “ Testosterone (T), the most abundant circulating androgen in males, is synthesised by the Leydig cells of the testes under the control of hypothalamus and anterior pituitary gland, and can further be converted to more potent form dihydrotestosterone (DHT) by the action of enzyme 5αR”
2. The comments for Table 1 (n **ow should be referred as Table 2**) regarding the unclarity due to missing of an indication of the phytochemicals’ relative efficacies has been addressed where the IC _50_ of each compound is included in the table and information on the type of cell lines used in each study is also added.
3. The suggestions made by the reviewer for the inclusion of another table highlighting the *in vivo* studies are appreciated and have been taken into consideration. We hereby added another table **(referred to now as Table 1)** that summarise the findings of anti-CaP studies of various phytochemicals, which also includes the *in vivo* studies.
4. The comments made by the reviewer in the Discussion section regarding the provision of additional context focusing on human clinical trials and future directions have now been addressed:
   
  - Discussion: The sentences “These findings are hoped to assist in the next stage of human clinical trials, as to date, only synthetic 5aRis are investigated in such setting. However, further isolation of these phytochemicals needs to be done especially from the plant sources before it can be implied in human clinical setting” is added. 

# Peer review: Reviewer response for version 1

**DOI:** 10.5256/f1000research.54175.r81614

**Published:** 2021-05-18

**Authors:** Binesh Shrestha

**Affiliations:** 1 Department of Chemical Biology and Therapeutics, Novartis Institutes For Biomedical Research, Basel, Switzerland

Introduction

- Paragraph 3:
  This can be simplified - e.g. in the second sentence, what does “limiting the production of androgen” mean? It would be clearer to say reduction or increase.
- Paragraph 4:
  Similar is true here. The content of the whole paragraph would be easier to follow if it is written in simple forms. One easy way would be to break down long sentences into short sentences.
- Paragraph 5:
  Please correct the typo mistakes of genus and species of some names - e.g. *Sebastiania *(genus starting with upper case) *adenophora *(species starting with lower case). Please correct the same in Table 1 as well for *Prunus africana *(throughout the article 3 times in Table 1 and also in text) and *Cocos nucifera.* For *Quercus acutissima *please change it to italics.

Discussion

- The third sentence starting with “The androgens, which are the.......as a part of the strategies” is very long (7 lines). Please simplify by breaking it down.

***The overall content is good and comprehensive. I believe that the researchers in the field will have a short comprehensive overview reflection from this review. With the above-mentioned corrections, I would recommend this review for indexing.***

Is the review written in accessible language?

Partly

Are all factual statements correct and adequately supported by citations?

Yes

Are the conclusions drawn appropriate in the context of the current research literature?

Yes

Is the topic of the review discussed comprehensively in the context of the current literature?

Yes

Reviewer Expertise:

Protein Biochemistry, Microbiology and Biotechnology

I confirm that I have read this submission and believe that I have an appropriate level of expertise to confirm that it is of an acceptable scientific standard, however I have significant reservations, as outlined above.

# Response

**Published:** 2021-05-22

**Authors:** Sheikh Naeem Shafqat

**Affiliations:** Universiti Brunei Darussalam, Brunei

Reply to the comments made by the Reviewer (Dr. Binesh Shrestha):

 Thank you for reviewing and suggesting some constructive amendments to the review article.

 Following are the actions taken in response to the reviewer’s comments. The reviewer should be able to see all the changes made to the article in response to the comments by downloading/reviewing the latest version of the review article.

  

- The comments made by the reviewer in Paragraph 3 and Paragraph 4 regarding the complexity of written text have been addressed and now it has been rewritten in a simple language suitable for the understanding of general readers.

 

- The comments made by the reviewer in Paragraph 5 regarding the few typo mistakes, while addressing some genus and species names, and the use of italic font has been addressed and all the corrections have been made.

 

- The comments made by the reviewer in the Discussion regarding the simplification of the written text by breaking down the text into smaller sentences, for the understanding of general readers have been addressed.

Thank You,

 Regards,

 Dr. Naeem Shafqat (Author of the article)
